# Association between hepatic steatosis and fibrosis and arthritis among US adults: A population-based study

**DOI:** 10.1016/j.clinsp.2024.100378

**Published:** 2024-06-13

**Authors:** Zhiming Lu, Shaojie Wu, Eryou Feng, Xiaoli Chen, Jinhua Chen, Feitai Lin

**Affiliations:** a Fujian Medical University Union Hospital, Fuzhou, China; b Fuzhou City Second Hospital, Fuzhou, China; c The Third Clinical Medical College, Fujian Medical University, China; d Fujian Provincial Clinical Medical Research Center for First 339 Aid and Rehabilitation in Orthopaedic Trauma (2020Y2014), China

**Keywords:** Hepatic steatosis, Liver fibrosis, Arthritis, Controlled attenuation parameter, NHANES

## Abstract

**Background:**

Lipid metabolism factors may play a role in the development of arthritis and hepatic steatosis and fibrosis. The aim of this study was to explore the potential association between arthritis and hepatic steatosis and liver fibrosis.

**Materials and methods:**

The nationally representative sample from the National Health and Nutrition Examination Survey was analyzed, with data on arthritis diagnosis, subtype, and liver status obtained. Liver status was assessed using transient elastography. Hepatic steatosis was defined as a Controlled Attenuation Parameter (CAP) score ≥263 dB/m, and liver fibrosis status was defined as F0‒F4. Logistic regression models and subgroup analyses stratified by sex were used to evaluate the associations. Smooth curve fitting was used to describe the associations.

**Results:**

The present study of 6,840 adults aged 20 years or older found a significant positive correlation between arthritis and CAP in multivariate logistic regression analysis (β = 0.003, 95 % CI 0.001 to 0.0041, *p* < 0.001). Participants with arthritis had a higher risk of hepatic steatosis (OR = 1.248, 95 % CI 1.036 to 1.504, *p* = 0.020), particularly those with osteoarthritis or degenerative arthritis, but not rheumatoid arthritis (*p* = 0.847). The positive correlation was maintained in females (β = 0.004, 95 % CI 0.002 to 0.006, *p* < 0.001), but not in males. There was no significant relationship between arthritis and liver fibrosis (*p* = 0.508).

**Conclusion:**

This study indicates that there is a positive correlation between arthritis and hepatic steatosis, particularly in females. Nonetheless, there is no significant relationship between arthritis and the risk of liver fibrosis.

## Highlights

• Positive correlation between arthritis and hepatic steatosis, particularly in women.
• No significant relationship between arthritis and the risk of liver fibrosis.
• First study to examine the association between arthritis and both liver steatosis and fibrosis.

## Introduction

Arthritis is an increasingly common disease with global implications for many health outcomes.^1^ According to Global Burden of Disease data, the age-standardized incidence rate of arthritis has a global annual growth rate of 0.32 % or an increase of approximately 9 % over a 28-year period.^2^ Recent studies have shown that metabolism plays a crucial role in arthritis, particularly in Osteoarthritis (OA), where metabolism undergoes significant changes^3^, ^4^, ^5^, ^6^ Wan-Su Choi et al.’s research in Nature indicated that OA is a disease associated with metabolic disorders.^7^ Previous research has indicated a relationship between arthritis patients and abnormal lipid metabolism factors such as cholesterol and low-density lipoprotein. Lipid abnormalities causing ectopic lipid deposition in chondrocytes may trigger the onset of osteoarthritis, while dysregulated cellular lipid metabolism in joint tissues may exacerbate osteoarthritis.^8^, ^9^, ^10^, ^11^

On the other hand, the liver plays a critical role in lipid metabolism, and abnormal lipid metabolism can lead to the development of liver steatosis, fibrosis, and even cirrhosis. This may be due to multiple functional impairments, such as very low-density lipoprotein secretion and pathways involved in fatty acid synthesis.^12^, ^13^, ^14^, ^15^ Metabolic dysfunction-associated fatty liver disease is highly prevalent, affecting approximately 25 % of the global population.^16^ Recently, non-invasive detection methods such as transient elastography (FibroScan®, TE) have been shown to be useful for screening for metabolic-related fatty liver disease. Controlled Attenuation Parameters (CAP) and Liver Stiffness Median (LSM) have demonstrated good accuracy in quantifying patients' liver fat and fibrosis levels.^17^^,^^18^

Currently, fatty liver and arthritis are two common chronic diseases with high incidence rates and global burdens. However, the association between fatty liver and arthritis has not been fully explored, and the metabolic-related pathogenesis of arthritis is still a controversial issue. Therefore, the aim of this study is to use a population-based sample from the National Health and Nutrition Examination Survey (NHANES) to investigate the association between CAP, LSM, as screening indicators of metabolic-related fatty liver disease, and arthritis in adults aged 20 and above, and further analyze the relationship between OA, Rheumatoid Arthritis (RA), and liver steatosis and fibrosis. To our knowledge, there is limited research that has investigated the relationship between hepatic steatosis, liver fibrosis, and arthritis using representative national cohorts.

## Materials and methods

### *Study population*

The data for this study were obtained from the National Health and Nutrition Examination Survey (NHANES 2017‒2020), including all the latest liver ultrasound Transient Elastography (TE) examination data. NHANES is a nationally representative cross-sectional study that uses a complex clustered, multistage, probability-based design for data collection and analysis, rather than a simple random sampling of the US population. Detailed information and characterization of the NHANES survey plan and design have been previously described.^19^^,^^20^ A total of 15,560 individuals completed the survey from 2017 ‒ March 2020. In this study, individuals with ages < 20 years (*n* = 6328), missing questionnaire information on osteoarthritis (*n* = 26), and those who did not complete the TE examination (*n* = 1829) were sequentially excluded. Additionally, one participant was excluded from the analysis due to the inability to obtain Controlled Attenuation Parameter (CAP) or Liver Stiffness Median (LSM) data. Finally, subjects with missing covariate data were excluded from the analysis (*n* = 536). A total of 6840 participants were included in the final study. NHANES is reviewed by the Ethics Review Committee of the National Center for Health Statistics, and all participants consented to the use of their anonymous information for research purposes.

### *Arthritis*

Medical condition questionnaires were administered through interviews as part of the NHANES to collect data on arthritis diagnoses. Participants aged 20 years and older were asked whether they had ever been told by a doctor or other health professional that they had arthritis. If so, they were asked to specify their arthritis type as osteoarthritis or degenerative arthritis, rheumatoid arthritis, psoriatic arthritis, other, or unknown/refusal. Based on their responses, participants were categorized into arthritis and non-arthritis groups. A previous study found that self-reported “definite” osteoarthritis had up to 81 % agreement with clinical confirmation suggesting that osteoarthritis is generally reported with a high level of accuracy.^21^

### *Hepatic steatosis and liver fibrosis*

Hepatic steatosis and liver fibrosis were measured using the FibroScan model 502 V2 Touch, which utilizes ultrasound and vibration-controlled transient elastography to derive liver stiffness and measure the ultrasound attenuation related to the presence of hepatic steatosis. The accuracy of transient elastography to assess liver steatosis and liver fibrosis has been evaluated in previous studies.^17^^,^^18^^,^^22^

The CAP and LSM were first used to evaluate hepatic steatosis and liver fibrosis in NHANES 2017 ‒ March 2020 participants. Only subjects who completed all tests (fasting time ≥ 3 h, complete stiffness tests ≥ 10 measures, and Interquartile Range [IQR] of liver stiffness/LSM < 30 %) were included in the current study. Combining the findings of Siddiqui,^23^ Eddowes,^24^ et al., hepatic steatosis was defined as CAP scores ≥ 263 dB/m without viral hepatitis (hepatitis B virus or hepatitis C virus infections). Non-Alcoholic Fatty Liver Disease (NAFLD) was diagnosed as the presence of hepatic steatosis without significant alcohol consumption (> 3 drinks/day in men and > 2 drinks/day in women).

According to the METAVIR grading system, liver fibrosis status was defined as Absent or mild fibrosis (F0‒F1), Significant fibrosis (F2), Severe fibrosis (F3), Cirrhosis (F4), with the cutoff values of LSM being 7.0, 9.5, and 12.5 (KPa), respectively. F2, F3, and F4 were considered to have clinically significant liver fibrosis.^25^

### *Covariates*

The study collected demographic data using interviews and questionnaires, including age, sex, Body Mass Index (BMI), race, education level, the ratio of family income to poverty, daily alcohol drinking status, physical activity level, and history of diabetes, hepatitis B and C. Age was classified into three categories: 20‒39 years, 40‒59 years, and over 60 years. BMI was classified as under/normal weight (< 25.0 kg/m^2^), overweight (25.0‒30.0 kg/m^2^), and obese (≥ 30.0 kg/m^2^). Race was quantified as non-Hispanic White, non-Hispanic Black, Mexican American, other Hispanic, and other races, including multiracial. The income-to-poverty ratio was classified into three categories: <1.3, 1.3‒1.8, and > 1.8. Education level was classified into three categories: more than high school, high school or equivalent, and less than high school. Daily alcohol drinking status was categorized as none, moderate (1 drink/day for women or 1‒2 drinks/day for men), heavy (2‒3 drinks/day for women or 3‒4 drinks/day for men), and binge (≥ 4 drinks/day for women or ≥ 5 drinks/day for men), based on the definitions by the National Institute on Alcohol Abuse and Alcoholism. Physical activity level was classified as active (≥ recommended levels of vigorous-intensity activity of at least 75 minutes per week or ≥ recommended levels of moderate-intensity activity of at least 150 minutes per week), inactive (below recommended levels), and no activity.^26^ The smoking level was categorized based on serum cotinine levels as low (< 0.015 ng/mL), moderate (0.015‒3 ng/mL), and high (> 3 ng/mL).^27^

### *Laboratory data*

The study collected biological samples for laboratory analysis to compare the nutritional status, liver function, lipid metabolism, and other detailed information between the arthritis group and the non-arthritis group. The biological specimens were gathered and processed at the mobile examination center before being transferred to the laboratory for subsequent analysis and storage. The following biomarkers were collected: alanine aminotransferase; alkaline phosphatase; aspartate aminotransferase; total bilirubin; total calcium; total cholesterol; uric acid; creatine phosphokinase; high-density lipoprotein; triglycerides; low-density lipoproteins and high-sensitivity C-reactive protein.

### *Statistical analysis*

The authors conducted a statistical analysis using STATA 16.0, graphical software R (version 4.1.3), and Empower Stats (version 2.0) in this study. To ensure national representativeness and reduce significant fluctuations in the dataset, the authors employed weighted methods recommended by NHANES analysis guidelines for complex survey design. The baseline characteristics of the study population were described and compared between arthritis and non-arthritis groups. Continuous variables were presented as weighted means ± SD and compared using weighted logistic regression. Categorical variables were presented as weighted percentages (95 % CI) and compared using chi-square tests. The association between liver elastography parameters (CAP, LSM) and arthritis, as well as the relationship between OA, RA hepatic steatosis and liver fibrosis based on population analysis, were calculated using multivariable logistic regression. Three models were constructed for multivariate analysis: Model 1 (unadjusted), Model 2 (adjusted for sex, age, and race), and Model 3 (adjusted for age, sex, race, poverty-income ratio, education level, daily alcohol drinking status, physical activity level, diabetes history, and hepatitis B history). To comply with the Strengthening the Reporting of Observational Studies in Epidemiology guidelines,^28^ the authors performed a subgroup analysis stratified by gender to better utilize the data. Finally, the authors conducted smooth curve fitting for statistically significant results by adjusting for variables. Statistical significance was considered when *p* < 0.05.

## Results

### *Study sample*

In this study, a total of 6840 adult participants were included based on the inclusion and exclusion criteria. The mean age of the participants was 48.01 ± 17.21 years, with 50.55 % being female and 49.45 % being male. Clinical characteristics of the participants were presented in Table 1, stratified by arthritis diagnosis. Compared to the non-arthritis group, the arthritis group was more likely to be female, older, and have lower education levels and lower household income, with a higher proportion of non-Hispanic white individuals. This study observed a higher prevalence of hepatic steatosis, NAFLD, and liver fibrosis in the arthritis group. There were statistically significant differences in LSM (5.99 ± 4.48 kPa vs. 5.61 ± 4.44 kPa) and CAP (275.84 dB/*m* ± 58.24 vs. 260.10±63.27 dB/m) between the non-arthritis group and the arthritis group (*p* < 0.05).

**Table 1:** Weighted demographic characteristics of study sample with and without arthritis.

	Arthritis (*n* = 2041)	Non-arthritis (*n* = 4799)	p-value
**Age (years)**	60.05 ± 13.71	43.54 ± 16.01	<0.001
Age groups (%)			<0.001
20‒39 years	9.26 (7.65, 11.16)	46.37 (44.22, 48.53)	
40‒59 years	35.3 (32.03, 38.71)	34.67 (32.62, 36.78)	
≥ 60 years	55.44 (52.08, 58.76)	18.96 (17.39, 20.64)	
**Gender (%)**			<0.001
Male	41.32 (38.07, 44.64)	52.47 (50.32, 54.61)	
Female	58.68 (55.36, 61.93)	47.53 (45.39, 49.68)	
**BMI Group (%)**	26.62 ± 8.52	26.37 ± 8.38	0.312
< 25	42.82 (39.59, 46.12)	43.1 (40.98, 45.24)	
25–30	26.92 (23.98, 30.07)	25.35 (23.53, 27.27)	
≥ 30	30.18 (27.26, 33.27)	31.51 (29.53, 33.57)	
Not recorded	0.08 (0.03, 0.24)	0.04 (0.01, 0.11)	
**Race (%)**			<0.001
Mexican American	4.5 (3.75, 5.4)	10.22 (9.34, 11.17)	
Other Hispanic	5.82 (4.88, 6.92)	8.26 (7.46, 9.15)	
Non-Hispanic White	70.81 (68.31, 73.19)	60.43 (58.55, 62.29)	
Non-Hispanic Black	10.35 (9.26, 11.56)	10.6 (9.84, 11.4)	
Other races ‒ including multi-racial	8.52 (7.07, 10.23)	10.49(9.61,11.43)	
**Education (%)**			0.016
More than high school	60.14 (56.88, 63.32)	63.72 (61.67, 65.73)	
High school or equivalent	28.89 (25.86, 32.12)	25.63 (23.75, 27.6)	
Less than high school	10.88 (9.45,12.49)	10.62 (9.68, 11.63)	
Not recorded	0.09 (0.03, 0.28)	0.03 (0.01, 0.1)	
**Poverty-income ratio (%)**			0.007
< 1.3	16.96 (14.92, 19.21)	15.25 (14.1, 16.48)	
1.3–1.8	9.17 (7.76, 10.8)	7.39 (6.61, 8.24)	
> 1.8	62.24 (59.18, 65.2)	66.4 (64.55, 68.2)	
Not recorded	11.64 (9.83, 13.73)	10.97 (9.79, 12.26)	
**Smoking (%) (serum cotinine levels, ng/mL)**			**0.159**
< 0.015	39.89 (36.68, 43.19)	37.99 (35.87, 40.16)	
0.015–3	35.34 (32.27, 38.52)	37.81 (35.79, 39.88)	
≥ 3	24.78 (21.97, 27.81)	24.20 (22.4, 26.08)	
**Daily alcohol drinking status (%)**			<0.001
Non-drinkers	5.11 (4.09, 6.37)	7.01 (6.07, 8.07)	
Moderate-drinkers	33.99 (30.83, 37.3)	33.75 (31.68, 35.88)	
Heavy-drinkers	21.21 (18.44, 24.26)	24.87 (23.04, 26.79)	
Binge-drinkers	18.96 (16.52, 21.65)	21.34 (19.61, 23.18)	
Not recorded	20.74 (18.44, 23.24)	13.04 (11.81, 14.37)	
**Physical activity level (%)**			<0.001
Inactive	47.27 (44, 50.57)	46.71 (44.56, 48.86)	
Less active	2.24 (1.41, 3.55)	1.84 (1.33, 2.53)	
Active	22.27 (19.53, 25.28)	26.84 (24.94, 28.83)	
Not recorded	28.21 (25.23, 31.39)	24.62 (22.83, 26.49)	
**History of diabetes (%)**			<0.001
Yes	18.59 (16.32, 21.09)	7.88 (6.86, 9.03)	
**Having HBV infection (%)**			<0.001
Yes	1.77 (1.03, 3.02)	0.71 (0.49, 1.03)	
**Having HCV infection (%)**			0.0619
Yes	2.27 (1.56, 3.3)	1.46 (1, 2.12)	
**Hepatic steatosis (%)**			<0.001
Yes	58.27 (54.97, 61.49)	46.67 (44.53, 48.83)	
**NAFLD (%)**			<0.001
Yes	22.66 (19.91, 25.67)	17.83 (16.30, 19.48)	<0.001
Liver fibrosis (%)			<0.001
Yes	17.11 (14.87, 19.61)	12.84 (11.46, 14.35)	
**Metavir F0‒F4 (%)**			0.001
Absent or mild fibrosis(F0‒F1)	82.89 (80.52, 83.70)	87.16 (85.65, 88.54)	
Significant fibrosis (F2)	10.11 (8.27, 12.30)	7.69 (6.62, 8.91)	
Severe fibrosis (F3)	3.39 (2.53, 4.54)	2.34 (1.80, 3.03)	
Cirrhosis (F4)	3.61 (2.71, 4.80)	2.81 (2.14, 3.69)	
**Which type of arthritis (%)**			
Osteoarthritis or degenerative arthritis	49.35		
Rheumatoid arthritis	15.74		
Psoriatic arthritis	1.51		
Other	11.31		
Not recorded	22.09		
**Transient Elastography**			
LSM (kPa)	5.99 ± 4.48	5.61 ± 4.44	0.002
CAP (dB/m)	275.84 ± 58.24	260.10 ± 63.27	<0.001
Waist Circumference (cm)	104.79 ± 15.99	98.45 ± 16.36	<0.001
**Laboratory parameters**			
ALT(U/L)	22.09 ± 16.58	23.31 ± 17.82	0.011
ALP(IU/L)	78.10 ± 25.02	73.65 ± 23.71	<0.001
AST(U/L)	21.66 ± 12.55	22.00 ± 12.93	0.329
Total bilirubin (umoL/L)	7.87 ± 4.32	8.19 ± 5.23	0.018
Total Calcium (mmoL/L)	2.33 ± 0.10	2.32 ± 0.09	<0.001
Uric acid (umoL/L)	322.84 ± 84.50	317.13 ± 84.46	0.013
Creatine Phosphokinase (IU/L)	136.16 ± 147.75	170.89 ± 342.08	<0.001
HSCRP (mg/L)	4.57 ± 10.00	3.34 ± 5.93	<0.001
Direct HDL-Cholesterol (mmoL/L)	1.42 ± 0.44	1.38 ± 0.40	<0.001
Total Cholesterol (mg/dL)	4.92 ± 1.07	4.83 ± 1.03	0.002
Triglycerides(mmoL/L)	1.35 ± 0.93	1.21 ± 0.87	<0.001
Low-Density Lipoproteins (mmoL/L)	2.89 ± 0.91	2.84 ± 0.90	0.160

Values are weighted mean ± SD or weighted% (95% Confidence Interval); p-values are weighted. Other races include American Indian or Alaska Native, Native Hawaiian or other Pacific Islanders, and multiracial persons. BMI, Body Mass Index; NAFLD, Non-Alcoholic Fatty Liver Disease; LSM, Liver Stiffness Median; CAP, Controlled Attenuation Parameter; HBV, Hepatitis B Virus; HCV, Hepatitis C Virus; ALT, Alanine Aminotransferase; ALP, Alkaline Phosphatase; AST, Aspartate Aminotransferase; HDL, High-Density Lipoprotein; HSCRP, High-Sensitivity C-Reactive Protein.

Additionally, laboratory indicators including alanine aminotransferase, alkaline phosphatase, total bilirubin, uric acid, direct high-density lipoprotein cholesterol, total cholesterol, creatine phosphokinase, and high-sensitivity C-reactive protein also showed statistically significant differences. Compared to participants without arthritis, individuals with arthritis exhibited lower levels of alanine aminotransferase, total bilirubin, and creatine phosphokinase. Conversely, higher levels were observed in the arthritis group for alkaline phosphatase, uric acid, direct high-density lipoprotein cholesterol, total cholesterol, waist circumference, triglycerides, and high-sensitivity C-reactive protein, among other indicators.

### *Association between arthritis and controlled attenuation parameter (CAP) or hepatic steatosis*

Table 2 summarizes the results of multivariate regression analyses. In the unadjusted model (β = 0.004, 95 % CI 0.003 to 0.005, *p* < 0.001), there was a strong correlation between arthritis and CAP. However, after adjusting for gender, age, and race variables, this significant positive correlation slightly decreased in Model 2 (β = 0.003, 95% CI 0.002 to 0.005, *p* < 0.001), but remained statistically significant. After adjusting for all covariates, the positive correlation between arthritis and CAP in Model 3 became β = 0.003, 95% CI 0.001 to 0.003, *p* < 0.001. Individuals with Osteoarthritis (OA) or Degenerative Arthritis (DA) had higher CAP values (β = 0.003, 95% CI 0.001 to 0.005, *p* = 0.005) compared to the non-arthritis group. After adjusting for covariates, there was no significant correlation between CAP and Rheumatoid Arthritis (RA).

**Table 2:** Associations between arthritis and Controlled Attenuation Parameter (CAP).

	Model 1, β (95% CI), p	Model 2, β (95% CI), p	Model 3, β (95% CI), p
**Non-arthritis**	**Reference**	**Reference**	**Reference**
Arthritis	0.004 (0.003, 0.005) <0.001	0.003 (0.002, 0.005) <0.001	0.003 (0.001, 0.004) <0.001
**Non-arthritis**	**Reference**	**Reference**	**Reference**
OA or DA	0.004 (0.002, 0.005) <0.001	0.003 (0.001, 0.005) 0.001	0.003 (0.001, 0.005) 0.005
RA	0.004 (0.001, 0.006) <0.001	0.002 (−0.001, 0.005) 0.061	0.002 (−0.001, 0.004) 0.157
**Stratified by gender**		**“Model 2″**	**“Model 3″**
Male			
Arthritis	0.003 (0.001, 0.005) 0.001	0.002 (−0.000, 0.004) 0.076	0.002 (−0.000, 0.004) 0.092
Female			
Arthritis	0.006 (0.005, 0.008) <0.001	0.004 (0.002, 0.006) <0.001	0.004 (0.002, 0.006) <0.001

Model 1 no covariates were adjusted; Model 2 age, gender, and race were adjusted; Model 3 age, gender, race, educational level, poverty-income ratio, daily alcohol drinking status, physical activity level, history of diabetes, hepatitis B virus were adjusted; “Model 2″ and “Model 3″ did not adjust for gender. OA, Osteoarthritis Arthritis; DA, Degenerative Arthritis; RA, Rheumatoid Arthritis.

**Fig. 1:**
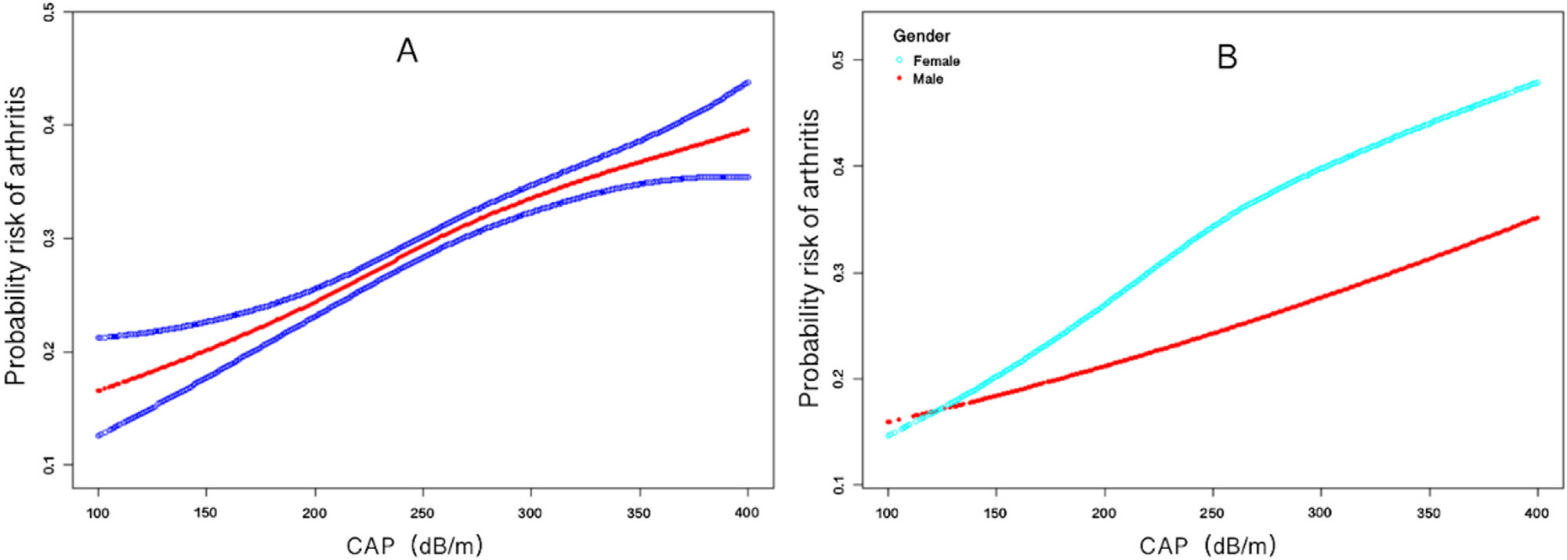
The association between arthritis and CAP. (A) The solid red line represents the smooth curve fit between variables. Blue bands represent the 95 % Confidence Interval from the fit. (B) Smoothed curve results stratified by gender. CAP, Controlled Attenuation Parameter.

In the subgroup analysis stratified by gender, the present results showed that the positive correlation between arthritis and CAP was independently and significantly positively associated with women (β = 0.004, 95% CI 0.002 to 0.006, *p* < 0.001), but not statistically significant in all adjusted models for men. The authors used a smoothing curve to describe the approximately linear relationship between arthritis and CAP. After stratifying by gender and adjusting for all covariates, both men and women showed a linear curve (Fig. 1).

Table 3 displayed the results of multivariate regression analyses that explored the association between arthritis and hepatic steatosis. In all models, participants with arthritis had a higher risk of hepatic steatosis than those without arthritis, and this association was statistically significant. The risk of hepatic steatosis was similar for participants with OA or DA compared to those with arthritis, but after adjusting for all covariates, this association became non-significant in Model 3 (OR = 1.256, 95% CI 0.977 to 1.614, *p* = 0.076). Participants with RA did not show a significant association with hepatic steatosis in any adjusted model (*p* = 0.847). In the gender-stratified subgroup analysis, women with OA or degenerative arthritis had a higher risk of hepatic steatosis than those without arthritis (OR = 1.866, 95% CI 1.409 to 2.470, *p* < 0.001). However, in the adjusted models for men, there was no significant association between hepatic steatosis and OA, DA or RA. The risk of hepatic steatosis was higher in women than in men.

**Table 3:** Associations between arthritis and hepatic steatosis.

	Model 1 OR (95% CI), p	Model 2, OR (95% CI), p	Model 3, OR (95% CI), p
**Non-arthritis**	**Reference**	**Reference**	**Reference**
Arthritis	1.595 (1.360, 1.872) <0.001	1.313 (1.094, 1.576) 0.003	1.248 (1.036, 1.504) 0.020
**Non-arthritis**	**Reference**	**Reference**	**Reference**
OA or DA	1.595 (1.279, 1.990) <0.001	1.304 (1.020, 1.670) 0.034	1.256 (0.977, 1.614) 0.076
RA	1.406 (1.030, 1.920) 0.032	1.133 (0.804, 1.595) 0.476	1.034 (0.735, 1.456) 0.847
**Stratified by gender**		**“Model 2″**	**“Model 3″**
Male			
OA or DA	1.610 (1.114, 2.329) 0.011	1.204 (0.812, 1.783) 0.355	1.166 (0.774, 1.756) 0.463
RA	1.353 (0.814, 2.249) 0.243	1.112 (0.642, 1.927) 0.705	1.042 (0.616, 1.763) 0.877
Female			
OA or DA	1.866 (1.409, 2.470) <0.001	1.369 (0.997, 1.878) 0.052	1.261 (0.912, 1.742) 0.161
RA	1.486 (1.033, 2.139) 0.033	1.116 (0.753, 1.655) 0.584	1.026 (0.672, 1.566) 0.906

Model 1 no covariates were adjusted; Model 2 age, gender, and race were adjusted; Model 3 age, gender, race, educational level, poverty-income ratio, daily alcohol drinking status, physical activity level, history of diabetes, hepatitis B virus were adjusted; “Model 2″ and “Model 3″ did not adjust for gender. OA, Osteoarthritis Arthritis; DA, Degenerative Arthritis; RA, Rheumatoid Arthritis.

### *Association between arthritis and liver stiffness median (LSM) or liver fibrosis*

**Fig. 2:**
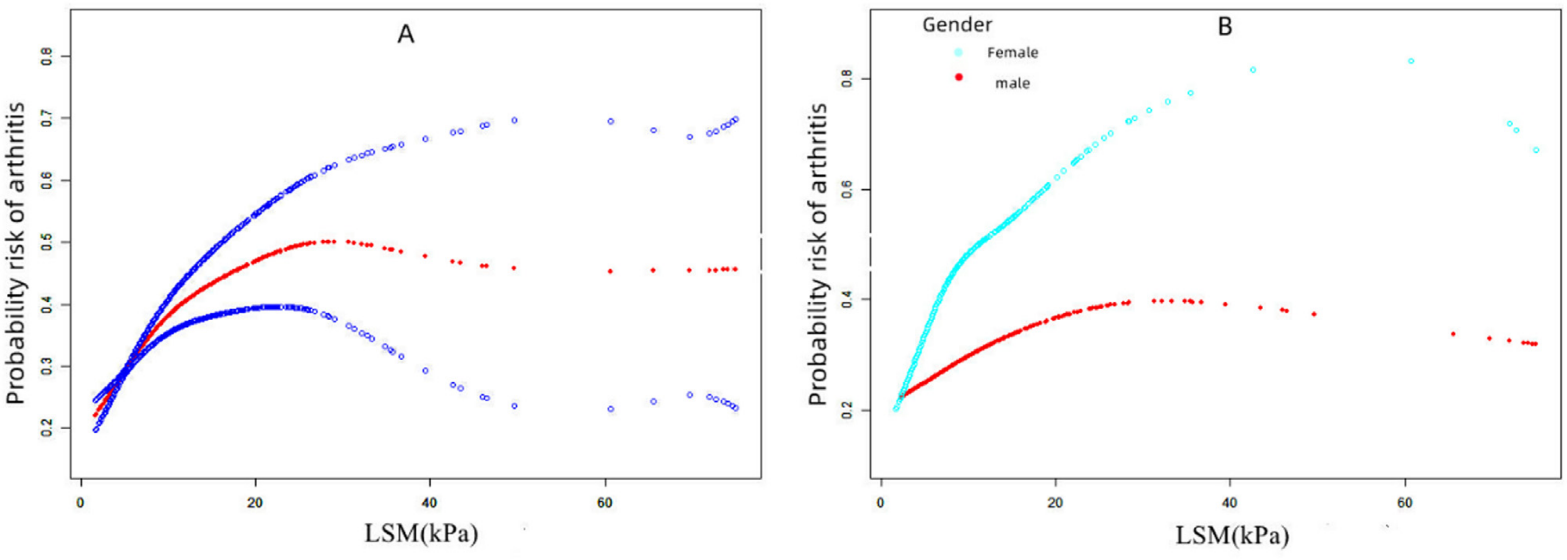
The association between arthritis and LSM. (A) The solid red line represents the smooth curve fit between variables. Blue bands represent the 95 % Confidence Interval from the fit. (B) Smoothed curve results stratified by gender. LSM, Liver Stiffness Median.

In Table 4, the results of multiple regression analysis showed a positive correlation between arthritis and LSM, but this correlation was not significant. However, in the gender-stratified subgroup analysis, the relationship between arthritis and LSM was significantly positively correlated in women (β = 0.030, 95% CI 0.003 to 0.058, *p* = 0.033), but not in men. In Table 5, patients with arthritis had a higher risk of liver fibrosis compared to those without arthritis, but this difference was not significant after adjusting for covariates. In the subgroup analysis, osteoarthritis or degenerative arthritis and rheumatoid arthritis were significantly positively correlated only in women without adjustment. The smoothing curve can be seen in Fig. 2.

**Table 4:** Associations between arthritis and Liver Stiffness Median (LSM).

	Model 1, β (95% CI), p	Model 2, β (95% CI), p	Model 3, β (95% CI), p
**Non-arthritis**	**Reference**	**Reference**	**Reference**
Arthritis	0.017 (0.002, 0.032) 0.032	0.013 (−0.002, 0.028) 0.093	0.007 (−0.008, 0.022) 0.349
**Non-arthritis**	**Reference**	**Reference**	**Reference**
OA or DA	0.013 (−0.005, 0.031) 0.158	0.011 (−0.008, 0.029) 0.261	0.006 (−0.013, 0.024) 0.565
RA	0.023 (0.006, 0.041) 0.007	0.018 (0.001, 0.035) 0.041	0.012 (−0.006, 0.029) 0.183
**Stratified by gender**		**“Model 2″**	**“Model 3″**
Male			
Arthritis	0.002 (−0.016, 0.018) 0.862	−0.008 (−0.027, 0.012) 0.426	−0.012 (−0.031, 0.008) 0.240
Female			
Arthritis	0.064 (−0.011, 0.140) 0.093	0.037 (0.004, 0.070) 0.026	0.030 (0.003, 0.058) 0.033

Model 1 no covariates were adjusted; Model 2 age, gender, and race were adjusted; Model 3 age, gender, race, educational level, poverty-income ratio, daily alcohol drinking status, physical activity level, history of diabetes, hepatitis B virus were adjusted; “Model 2″ and “Model 3″ did not adjust for gender. OA, Osteoarthritis Arthritis; DA, Degenerative Arthritis; RA, Rheumatoid Arthritis.

**Table 5:** Associations between arthritis and liver fibrosis.

	Model 1 OR (95% CI), p	Model 2, OR (95% CI), p	Model 3, OR (95%CI), p
**Non-arthritis**	**Reference**	**Reference**	**Model 3,**
arthritis	1.401 (1.134, 1.730) 0.002	1.189 (0.936, 1.510) 0.157	1.086 (0.851, 1.385) 0.508
**Non-arthritis**	**Reference**	**Reference**	**Reference**
OA or DA	1.271 (0.949, 1.702) 0.108	1.082 (0.781, 1.501) 0.635	1.013 (0.720, 1.426) 0.941
RA	1.461 (0.949, 2.248) 0.085	1.208 (0.777, 1.876) 0.401	1.057 (0.706, 1.583) 0.789
**Stratified by gender**		**“Model 2″**	**“Model 3″**
Male			
OA or DA	1.221 (0.780, 1.911) 0.382	0.995 (0.612, 1.618) 0.983	0.954 (0.570, 1.597) 0.859
RA	1.234 (0.630, 2.418) 0.540	1.079 (0.549, 2.120) 0.826	0.906 (0.509, 1.612) 0.736
Female			
OA or DA	1.617 (1.086, 2.409) 0.018	1.147 (0.730, 1.803) 0.553	1.057 (0.663, 1.686) 0.815
RA	1.891 (1.168, 3.061) 0.010	1.365 (0.822, 2.268) 0.230	1.282 (0.752, 2.183) 0.361

Model 1 no covariates were adjusted; Model 2 age, gender, and race were adjusted; Model 3 age, gender, race, educational level, poverty-income ratio, daily alcohol drinking status, physical activity level, history of diabetes, hepatitis B virus were adjusted; “Model 2″ and “Model 3″ did not adjust for gender. OA, Osteoarthritis Arthritis; DA, Degenerative Arthritis; RA, Rheumatoid Arthritis.

## Discussion

The present study analyzed nationally representative sample data of adult Americans and found that higher levels of CAP and LSM were positively associated with arthritis in women, but not in men. Among the subtypes of arthritis, osteoarthritis or degenerative arthritis was significantly associated with CAP, but not with LSM, while rheumatoid arthritis showed no significant association with either CAP or LSM. Furthermore, individuals with arthritis had a statistically significant increased risk of hepatic steatosis, but not liver fibrosis. It is worth noting that to our knowledge, this is the first study to examine the association between arthritis and both liver steatosis and fibrosis since the proposed pathogenesis of lipid metabolism in arthritis. This finding has significant clinical implications.

Based on epidemiological and basic research, the authors have recognized the impact of metabolic factors on arthritis. In 2009, Puenpatom et al..^29^ analyzed the NHANES III database and demonstrated an increased prevalence of metabolic syndrome in patients with osteoarthritis. Recent studies have shown that metabolic disturbances caused by adipose tissue-derived inflammatory mediators (adipokines), dyslipidemia, hyperglycemia, insulin resistance, or dyslipidemia can lead to joint metabolism disorder.^30^ Cholesterol levels in serum have been associated with the occurrence of osteoarthritis. Inflammatory markers such as C-reactive protein associated with high cholesterol levels may worsen the symptoms and severity of arthritis.^31^^,^^32^ Hotamisligil et al..^33^^,^^34^ described this type of inflammation as metabolic inflammation, and they found that obese mice released more Tumor Necrosis Factor (TNF) in adipose tissue, resulting in poor insulin sensitivity and glucose homeostasis, thus linking inflammation with metabolic disorders. In the present study, the arthritis group had higher serum levels of metabolic markers such as total cholesterol than the non-arthritis group, consistent with previous studies. Liver fat accumulation is often caused by excessive accumulation of adipose tissue in the liver and is a pathological change associated with metabolic abnormalities. This may be one of the reasons why participants with arthritis in this analysis had an increased risk of liver fat accumulation. At the same time, lipids are one of the important sources of nutrition for chondrocytes and may specifically affect cartilage formation and synovial inflammation through the action of oxidized Low-Density Lipoprotein (ox-LDL).^5^^,^^11^

From simple hepatic steatosis, it can progress to severe liver fibrosis and cirrhosis through leukocyte infiltration and hepatocyte ballooning.^35^ The mechanism underlying the link between inflammation and the progression of liver fibrosis and cirrhosis is not yet clear. Liver fibrosis is generally defined as the net deposition of extracellular matrix resulting from liver damage caused by various etiologies such as viral hepatitis and nonalcoholic fatty liver disease.^13^ Liver fibrosis begins with the recruitment of inflammatory immune cells that produce cytokines and other activating molecules, which in turn drive the production of ECM components by activated Hepatic Stellate Cells (HSCs) via chemical mediators. However, a study of NAFLD patients showed that steatosis, ballooning, and lobular inflammation were independently associated with significant fibrosis, and one-third of patients with significant fibrosis did not have Nonalcoholic Steatohepatitis (NASH), which was unexpected.^36^ Ruijie^37^ et al. similarly found no association between systemic immune-inflammation and liver fibrosis. In the present results, the authors found a lack of significant association between arthritis and liver fibrosis, supporting this phenomenon. This may be due to arthritis being a chronic inflammation that primarily involves joint cartilage, synovium, ligaments, muscles, and bone tissues surrounding the joint, leading to insulin resistance and subsequent accumulation of fat in the liver, but its “metabolic inflammation” is often a low-grade systemic inflammation, which is not consistent with the necrotic inflammation of liver fibrosis or even cirrhosis. This involves a series of mechanisms, such as increased oxidative stress, and accumulation of advanced glycation end products or free fatty acids, and the specific mechanisms still need further exploration.^38^^,^^39^ On the other hand, this may be due to a relatively small number of individuals with fibrosis in our sample.

In the present study, the gender-specific differences in arthritis, hepatic steatosis, and CAP are intriguing. Previous research has also reported gender-specific differences between chronic liver disease and various metabolic diseases such as obesity and diabetes. Visceral fat tissue accumulation in men and postmenopausal women increases faster with age and weight compared to young women. Furthermore, in postmenopausal women, the distribution of body fat shifts towards visceral fat, which may be due to their sex hormones.^12^^,^^38^^,^^40^ Age and gender are independent risk factors for osteoarthritis, with postmenopausal women being more susceptible to osteoarthritis, which may be related to a decrease in bone density.^41^ Current research indicates that endogenous estrogen has a protective effect on non-alcoholic fatty liver disease and bone density,^41^^,^^42^ which may explain why female arthritis participants are at a relatively higher risk for hepatic steatosis compared to males. However, Formyl Peptide Receptor 2 (FPR2) has a protective effect on the liver, and a recent animal experiment found that FPR2 expression in female mice is higher than in male mice, making females more resistant to developing hepatic steatosis and liver fibrosis.^43^ A study on non-alcoholic fatty liver disease in Korean adults found that males with NAFLD had more severe hepatic steatosis than females.^44^ Differences between studies may be attributed to differences in population demographics, sample size, study design, and controlled confounding variables.

Non-invasive markers have become indispensable tools in epidemiological research for assessing the prevalence of NAFLD. However, it has been recognized that these markers often underestimate the actual disease burden. In light of this limitation, studies employing non-invasive diagnostic techniques, such as Transient Elastography (TE), have emerged as promising approaches to enhance the accuracy of NAFLD diagnosis. The inclusion of TE in the present study serves to strengthen the credibility and validity of these findings.^45^ The present study has several main limitations. Firstly, this was a cross-sectional analysis and causality cannot be determined. In addition, although several relevant confounding factors were adjusted for, the authors cannot exclude the influence of other confounding factors. TE may be the most effective noninvasive method for assessing liver stiffness, but there is no histological confirmation. Despite these limitations, this study still has several strengths. The authors used a nationally representative sample, the large sample size included in the present study allowed us to conduct subgroup analyses. In addition, as the first study to explore the significant positive correlation between arthritis and hepatic steatosis and liver fibrosis, the authors provided epidemiological evidence for the metabolic correlation of arthritis. Future studies should explore the potential mechanisms underlying these differences to better understand the pathophysiology. In summary, the present study suggests that arthritis patients, particularly women, may have an increased risk of hepatic steatosis. This finding emphasizes the importance of monitoring liver health in arthritis patients, particularly those with risk factors for liver disease. Further research is needed to confirm these findings and explore the potential mechanisms between arthritis and liver disease. highlighted.

## Conclusions

There is a significant positive correlation between arthritis and hepatic steatosis, particularly in females, which may increase the risk of hepatic steatosis. However, no significant association was observed between arthritis and liver fibrosis risk. To confirm the present findings, more large-scale prospective investigations are needed.

## Ethics statement

The NHANES study procedures were reviewed and approved by the NCHS Ethics Review Board. Informed consent was obtained from all subjects involved in NHANES.

## Authors’ contributions

ZL, SW, EF: Conceptualization; Methodology; Software. ZL, SW, XC: Data curation; Writing-Original draft preparation. JC, FL: Visualization; Investigation. EF: Supervision. EF: Writing-Reviewing and Editing.

## Funding

This research received no external funding.

## Declaration of competing interest

The authors declare that the research was conducted in the absence of any commercial or financial relationships that could be construed as a potential conflict of interest.

## Data Availability

The datasets presented in this study can be found in online repositories (https://www.cdc.gov/nchs/nhanes/index.htm).

## References

[1] Park J., Mendy A., Vieira E.R. (2018). Various Types of Arthritis in the United States: prevalence and Age-Related Trends From 1999 to 2014. Am J Public Health.

[2] Quicke J.G., Conaghan P.G., Corp N., Peat G. (2022). Osteoarthritis year in review 2021: epidemiology & therapy. Osteoarthritis Cartilage.

[3] Zhuo Q., Yang W., Chen J., Wang Y. (2012). Metabolic syndrome meets osteoarthritis. Nat Rev Rheumatol.

[4] Mobasheri A., Matta C., Zákány R., Musumeci G. (2015). Chondrosenescence: definition, hallmarks and potential role in the pathogenesis of osteoarthritis. Maturitas.

[5] Mobasheri A., Rayman M.P., Gualillo O., Sellam J., van der Kraan P., Fearon U. (2017). The role of metabolism in the pathogenesis of osteoarthritis. Nat Rev Rheumatol.

[6] Papathanasiou I., Anastasopoulou L., Tsezou A. (2021). Cholesterol metabolism related genes in osteoarthritis. Bone.

[7] Choi W.-S., Lee G., Song W.-H., Koh J.-T., Yang J., Kwak J.-S. (2019). The CH25H-CYP7B1-RORα axis of cholesterol metabolism regulates osteoarthritis. Nature.

[8] Zheng L., Zhang Z., Sheng P., Mobasheri A. (2021). The role of metabolism in chondrocyte dysfunction and the progression of osteoarthritis. Ageing Res Rev.

[9] Cao C., Shi Y., Zhang X., Li Q., Zhang J., Zhao F. (2022). Cholesterol-induced LRP3 downregulation promotes cartilage degeneration in osteoarthritis by targeting Syndecan-4. Nat Commun.

[10] González-Gay M.A., González-Juanatey C (2014). Inflammation and lipid profile in rheumatoid arthritis: bridging an apparent paradox. Ann Rheum Dis.

[11] Yang Y., Wei J., Li J., Cui Y., Zhou X., Xie J. (2021). Lipid metabolism in cartilage and its diseases: a concise review of the research progress. Acta Biochim Biophys Sin (Shanghai).

[12] Mato J.M., Alonso C., Noureddin M., Lu S.C (2019). Biomarkers and subtypes of deranged lipid metabolism in non-alcoholic fatty liver disease. World J Gastroentero.

[13] Loomba R., Friedman S.L., Shulman G.I. (2021). Mechanisms and disease consequences of nonalcoholic fatty liver disease. Cell.

[14] Di Costanzo A., D'Erasmo L., Polimeni L., Baratta F., Coletta P., Di Martino M. (2017). Non-alcoholic fatty liver disease and subclinical atherosclerosis: a comparison of metabolically- versus genetically-driven excess fat hepatic storage. Atherosclerosis.

[15] Vergani L. (2019). Fatty Acids and Effects on In Vitro and In Vivo Models of Liver Steatosis. Curr Med Chem.

[16] Eslam M., Newsome P.N., Sarin S.K., Anstee Q.M., Targher G., Romero-Gomez M. (2020). A new definition for metabolic dysfunction-associated fatty liver disease: an international expert consensus statement. J Hepatol.

[17] Tang A., Cloutier G., Szeverenyi N.M., Sirlin C.B. (2015). Ultrasound Elastography and MR Elastography for Assessing Liver Fibrosis: part 1, Principles and Techniques. AJR Am J Roentgenol.

[18] Barr R.G., Ferraioli G., Palmeri M.L., Goodman Z.D., Garcia-Tsao G., Rubin J. (2015). Elastography Assessment of Liver Fibrosis: society of Radiologists in Ultrasound Consensus Conference Statement. Radiology.

[19] Kan B., Guo D., Yuan B., Vuong A.M., Jiang D., Zhang M. (2021). Dietary carotenoid intake and osteoporosis: the National Health and Nutrition Examination Survey, 2005-2018. Arch Osteoporos.

[20] Akinbam L., Chen T.-C., Davy O., Ogden C.L., Fink S., Clark J. (2022). National Health and Nutrition Examination Survey, 2017–March 2020 Prepandemic File: sample Design, Estimation, and Analytic Guidelines. Vital Health Stat.

[21] March L.M., Schwarz J.M., Carfrae B.H., Bagge E. (1998). Clinical validation of self-reported osteoarthritis. Osteoarthritis Cartilage.

[22] Jiang W., Huang S., Teng H., Wang P., Wu M., Zhou X. (2018). Diagnostic accuracy of point shear wave elastography and transient elastography for staging hepatic fibrosis in patients with non-alcoholic fatty liver disease: a meta-analysis. BMJ Open.

[23] Siddiqui M.S., Vuppalanchi R., Van Natta M.L., Hallinan E., Kowdley K.V., Abdelmalek M. (2019). Vibration-Controlled Transient Elastography to Assess Fibrosis and Steatosis in Patients With Nonalcoholic Fatty Liver Disease. Clin Gastroenterol Hepatol.

[24] Eddowes P.J., Sasso M., Allison M., Tsochatzis E., Anstee Q.M., Sheridan D. (2019). Accuracy of FibroScan Controlled Attenuation Parameter and Liver Stiffness Measurement in Assessing Steatosis and Fibrosis in Patients With Nonalcoholic Fatty Liver Disease. Gastroenterology.

[25] Castera L., Forns X., Alberti A. (2008). Non-invasive evaluation of liver fibrosis using transient elastography. J Hepatol.

[26] Piercy K.L., Troiano R.P., Ballard R.M., Carlson S.A., Fulton J.E., Galuska D.A. (2018). The Physical Activity Guidelines for Americans. JAMA.

[27] Akinkugbe A.A., Moreno O., Brickhouse T.H. (2019). Serum cotinine, vitamin D exposure levels and dental caries experience in U.S. adolescents. Community Dent Oral Epidemiol.

[28] Vandenbroucke J.P., von Elm E., Altman D.G., Gøtzsche P.C., Mulrow C.D., Pocock S.J. (2007). Strengthening the Reporting of Observational Studies in Epidemiology (STROBE): explanation and elaboration. PLoS Med.

[29] Puenpatom R.A., Victor T.W. (2009). Increased prevalence of metabolic syndrome in individuals with osteoarthritis: an analysis of NHANES III data. Postgrad Med.

[30] Griffin T.M., Huebner J.L., Kraus V.B., Yan Z., Guilak F. (2012). Induction of osteoarthritis and metabolic inflammation by a very high-fat diet in mice: effects of short-term exercise. Arthritis Rheum.

[31] Semb A.G., Ikdahl E., Wibetoe G., Crowson C., Rollefstad S. (2020). Atherosclerotic cardiovascular disease prevention in rheumatoid arthritis. Nat Rev Rheumatol.

[32] Zhang M., Deng Q., Wang L., Huang Z., Zhou M., Li Y. (2018). Prevalence of dyslipidemia and achievement of low-density lipoprotein cholesterol targets in Chinese adults: a nationally representative survey of 163,641 adults. Int J Cardiol.

[33] Hotamisligil G.S. (2006). Inflammation and metabolic disorders. Nature.

[34] Hotamisligil G.S., Shargill N.S., Spiegelman B.M. (1993). Adipose expression of tumor necrosis factor-alpha: direct role in obesity-linked insulin resistance. Science.

[35] Long M.T., Zhang X., Xu H., Liu C.-T., Corey K.E., Chung R.T. (2021). Hepatic fibrosis associates with multiple cardiometabolic disease risk factors: the framingham heart study. Hepatology.

[36] Pelusi S., Cespiati A., Rametta R., Pennisi G., Mannisto V., Rosso C. (2019). Prevalence and Risk Factors of Significant Fibrosis in Patients With Nonalcoholic Fatty Liver Without Steatohepatitis. Clin Gastroenterol Hepatol.

[37] Xie R., Xiao M., Li L., Ma N., Liu M., Huang X. (2022). Association between SII and hepatic steatosis and liver fibrosis: a population-based study. Front Immunol.

[38] Friedman S.L., Neuschwander-Tetri B.A., Rinella M., Sanyal A.J (2018). Mechanisms of NAFLD development and therapeutic strategies. Nat Med.

[39] Sanyal A.J. (2019). Past, present and future perspectives in nonalcoholic fatty liver disease. Nat Rev Gastroenterol Hepatol.

[40] Kerekes G., Nurmohamed M.T., González-Gay M.A., Seres I., Paragh G., Kardos Z. (2014). Rheumatoid arthritis and metabolic syndrome. Nat Rev Rheumatol.

[41] Maeda S.S., Lazaretti-Castro M. (2014). An overview on the treatment of postmenopausal osteoporosis. Arq Bras Endocrinol Metabol.

[42] Palmisano B.T., Zhu L., Stafford J.M. (2017). Role of estrogens in the regulation of liver lipid metabolism. Adv Exp Med Biol.

[43] Lee C., Kim J., Han J., Oh D., Kim M., Jeong H. (2022). Formyl peptide receptor 2 determines sex-specific differences in the progression of nonalcoholic fatty liver disease and steatohepatitis. Nat Commun.

[44] Lee H., Lee Y.-H., Kim S.U., Kim H.C. (2021). Metabolic dysfunction-associated fatty liver disease and incident cardiovascular disease risk: a nationwide cohort study. Clin Gastroenterol Hepatol.

[45] Xie R., Zhang Y. (2023). Is assessing the degree of hepatic steatosis and fibrosis based on index calculations the best choice for epidemiological studies?. Environ Pollut.

